# DIMASS: A Delaunay-Inspired, Hybrid Approach to a Team of Agents Search Strategy

**DOI:** 10.3389/frobt.2022.851846

**Published:** 2022-06-29

**Authors:** Sagir M. Yusuf, Chris Baber

**Affiliations:** School of Computer Science, University of Birmingham, Birmingham, United Kingdom

**Keywords:** unmanned aerial vehicle, area coverage path planning, multi-agent searching, constraint optimization, distributed constraints optimization, team of UAVs search

## Abstract

This article describes an approach for multiagent search planning for a team of agents. A team of UAVs tasked to conduct a forest fire search was selected as the use case, although solutions are applicable to other domains. Fixed-path (e.g., parallel track) methods for multiagent search can produce predictable and structured paths, with the main limitation being poor management of agents’ resources and limited adaptability (i.e., based on predefined geometric paths, e.g., parallel track, expanding square, etc.). On the other hand, pseudorandom methods allow agents to generate well-separated paths; but methods can be computationally expensive and can result in a lack of coordination of agents’ activities. We present a hybrid solution that exploits the complementary strengths of fixed-pattern and pseudorandom methods, i.e., an approach that is resource-efficient, predictable, adaptable, and scalable. Our approach evolved from the Delaunay triangulation of systematically selected waypoints to allocate agents to explore a specific region while optimizing a given set of mission constraints. We implement our approach in a simulation environment, comparing the performance of the proposed algorithm with fixed-path and pseudorandom baselines. Results proved agents’ resource utilization, predictability, scalability, and adaptability of the developed path. We also demonstrate the proposed algorithm’s application on real UAVs.

## 1 Introduction

**FIGURE 1:**
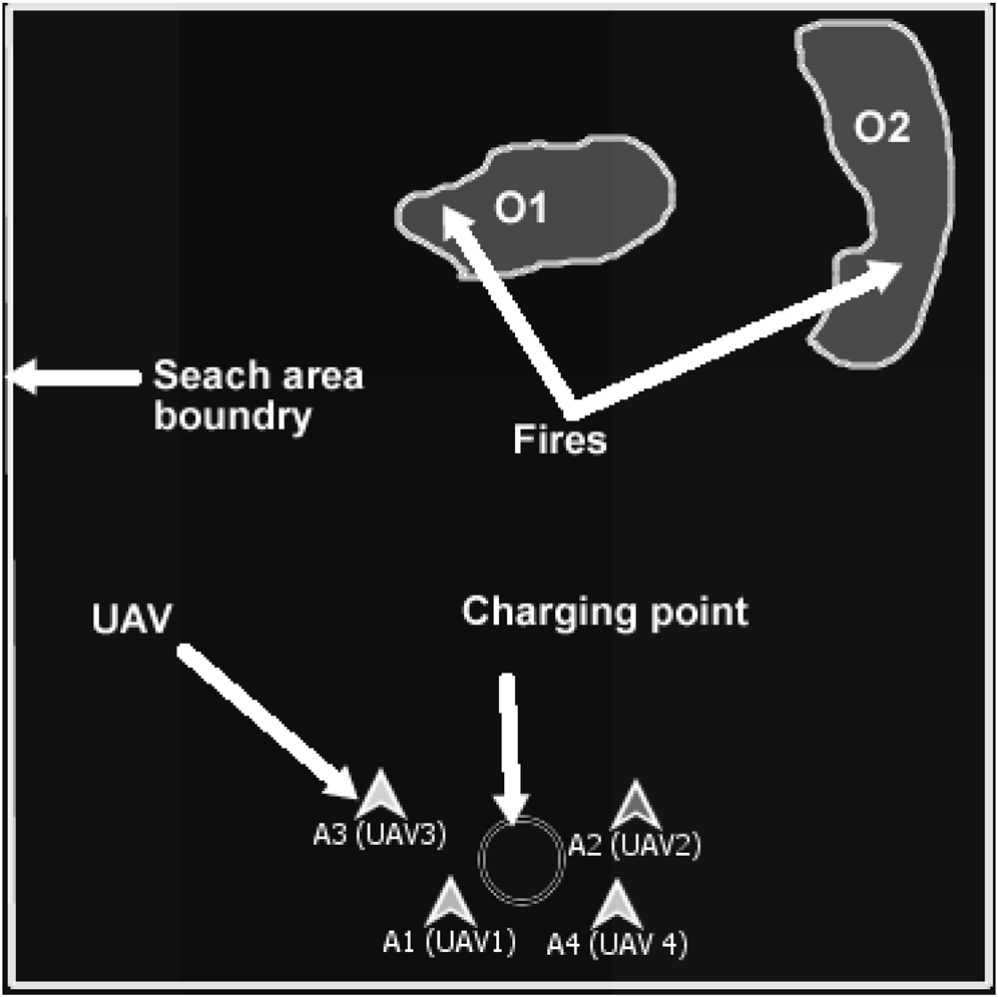
Example of multi-UAV area coverage problem. A1–A4 are UAVs that are tasked to search forest fires O1 and O2.

The objective of multiagent planning (MAP) for search is to coordinate the activities of agents to explore an area of interest and detect prescribed targets while optimizing relevant parameters (Cabreira et al., 2019; Nebel et al., 2019). For example, in Figure 1, A1–A4 are unmanned aerial vehicles (UAVs) that have been assigned the mission of exploring a search area (bounded by defined perimeters, i.e., the rectangular border) in order to detect forest fires (O1 and O2) which will be moving across the search area. The forest fires’ movement depends on variables such as wind speed, wind direction, fuel type, and buildings, i.e., the environment is dynamic. In our motivating use case of a mission for forest searching (Figure 1), cost functions are associated with limitations of sensor range, energy (power or battery capacity of the agents), agents’ interactions, communication range, onboard computational power, and memory use. Thus, our version of multiagent search is a team of agents (e.g., UAVs) tasked to conduct a search activity under the outlined constraints.

In addition, individual agents do not know the locations of the targets (fires), and the location of targets may change due to exogenous events, e.g., the forest fires O1 and O2 in Figure 1 move faster downwind proportional to wind speed and fuel type. Each agent is responsible for implementing its local plan (i.e., exploring a sequence of waypoints). The UAVs’ mission is to explore the search area and find the fires through effective resources (e.g., battery and mission time, as outlined in Table 1) utilization, efficient agent coordination (by avoiding redundant searches), and satisfying the imposed constraints (e.g., agents’ sparse interactions). This is a challenging problem because the agents need to ensure coverage of a dynamic environment with as little redundancy as possible while optimizing a set of mission constraints. This motivating use case mimics many practical applications such as cases of a missing person finding, agents mapping, search and rescue, and disaster management. (Bevacqua et al., 2015; Cooper, 2020; Drew, 2021; Heintzman et al., 2021; Nguyen et al., 2021; Sadat et al., 2014; Setter and Egerstedt, 2017). Our use case assumes a number of simple agents (e.g., micro-UAVs in Table 3) carrying targets’ detection sensors (e.g., infrared sensors, temperature sensors, and spectrum cameras for fire detection, on the assumption of one UAV per sensor) and reports their information to the picture compilers (PCs) UAVs (high-capacity fixed-wing or multirotor UAVs). PCs are responsible for simple agents’ data processing. The PCs will then submit their information to a server (a host computer responsible for combined system knowledge processing).

**TABLE 1:** Agent and mission parameters to be optimiszd.

Parameter	Optimization	Parameter type
Energy (battery) use	Minimize	Agent
Memory use	Minimize	Agent
Computational power	Minimize	Agent
Communication range	Minimize	Agent
Coverage	Maximize	Mission
Path divergence (path separation in search space)	Maximize	Mission
Redundant search	Minimize during search	Mission
Mission time	Minimize	Mission

Common MAP search methods will either assign fixed paths for each agent in the team or enable individual agents to adapt their actions to the environment and can operate in a centralized or decentralized manner (Cabreira et al., 2019, 2018; Corte et al., 2020; Merino et al., 2006, 2010; Ghamry and Zhang, 2016). These are discussed further in Section 2. There are also “hybrid” methods that enable agents to use a combination of fixed and adaptive protocols (Chawla and Duhan, 2018; Cabreira et al., 2019, 2018; Jensen-Nau et al., 2021). Given the constraints imposed by our motivating example, we contribute to the set of hybrid methods, with an emphasis on supporting search under resource constraints. To be specific, our approach

• Builds on a Delaunay triangulation of the search space to allocate agents to regions while optimizing resources;
• Provides a structured method for the team of agent search;
• Supports scalability, adaptability, and predictability;

We implement our approach on a small team of UAVs in the Aerospace Multi-Agent Simulation Environment (AMASE) developed by the Aerospace Vehicle Technology Assessment and Simulation Branch of the United States Air Force Research Laboratories (AFRL).^1^ AMASE models the environment and its elements, e.g., fire, wind speed, etc., UAVs and their parameters, sensors, and agents’ communication using MVC (Model-View-Controller). The AMASE views (e.g., fires, forests, etc.) can be designed using eXtensible Markup Language (XML) elements or controller languages such as Java and Python. The view elements can be referenced using unique identification numbers or names. Dynamic variables can be modeled using time-based controller functions. A detailed explanation of the simulation procedure and sample code can be found in Section 4 and supplemental documents. Section 6 describes a step-by-step procedure for applying the proposed solution to real UAVs (drones). We compare our approach with examples of the fixed path and pseudorandom baseline methods to evaluate resource utilization, predictability, adaptability, and scalability in a defined multi-UAVs mission for forest fire searching.

### 1.1 Problem Formulation

The focus of this article is on multiagent searching under resource constraints.

#### 1.1.1 Defining Resource Constraints

The resources (mission and agent parameters) of interest are summarized in Table 1. The choice of the parameters is in line with the literature (Cabreira et al., 2018, 2019; Bevacqua et al., 2015; Merino et al., 2010; Ingle, 2011). The resource parameters are the main limitation of applying UAVs to the search problems (Cabreira et al., 2018, 2019; Ghamry and Zhang, 2016; Jensen-Nau et al., 2021; Kanistras et al., 2013; Sharma and Kumar, 2015; Zhou et al., 2020; Alyassi et al., 2021; Rebolledo et al., 2021; Ryoo et al., 2010). The limitations in these parameters depend on the UAVs’ capability as described by the United Kingdom Ministry of Defence UAVs classification^2^ of Table 2. For our use case, micro or mini UAV types will be used. The parameters for the search mission modeled in this article are defined as cost functions (an example of how these are quantified is shown in Table 3) to enable the calculation of optimized solutions.

**TABLE 2:** UAV classification adapted.

UAV Classification	Maximum take off weight
Nano	< 200 g
Micro	> 200g–2 kg
Mini	2–20 kg
Small	> 20–150 kg
Medium	> 150–600 kg
Large	> 600 kg

**TABLE 3:** Example of cost functions.

Distance (D) between waypoints in kilometers square **(*km*** ^**2**^ **)**	Coverage cost (R)	Redundant search cost (R)	Mission time (R)
D $<1km^{2}$	2	20	20
1* *km^2^ ≤ *D* ≤ 2* *km^2^	10	10	10
2* *km^2^ ≤ *D* ≤ 3* *km^2^	20	2	2

#### 1.1.2 MAP/DULAR

The multiagent search problem developed in this article is referred to as the “MultiAgent Planning under Destination Uncertainty and Limited Resources (MAP/DULAR) problem”. We modeled MAP/DULAR as a finite-horizon, proactive, dynamic, and multi-objective distributed constraint optimization problem, PDMO-DCOP, defined by the tuple:

$$D=\left\{A_{i,j},T,W,\lambda ,P,\alpha_{i},\gamma_{i},\delta ,S_{\mathrm{condition}},K_{i},I,C,O,S\right\},$$ (1)

where

• *A*_*ij*_ = {*a*_11_, *a*_22_, *a*_33_, …, *a*_*ij*_} is the set of agents i of type j, *i* ∈ [1, *N*], *j* ∈ [1, *M*]. For example, a fire detecting micro-UAV of type simple agent and a fire understanding UAV of type PC.
• T is the mission time space *T*_*i*_ = *t*_1_, *t*_2_, *t*_3_, …, *t*_*i*_ for *i* = 1, …, *N*. This defines the mission activities with a finite horizon (Hoang et al., 2017; Fioretto et al., 2018) and can be measured using the mission clock.
• W are the paths (sequence of waypoints) for the agents, *W* = *w*_1_, *w*_2_, *w*_3_, …, *w*_*N*_, which are DCOP variables.
• *γ*_*i*_ is the set of agent’s situations (defined by the agent’s current belief about the environment, i.e., sensor states and location) over time period T, i.e., *γ*_*i*_ = {*γ*_1_ × *γ*_2_ × *γ*_3_ × ... × *γ*_*N*_}. For example, an agent situation can be fire presence (based on the sensor state) at location *w*_*i*_.
• *α*_*i*_ is the set of action spaces at every agent’s situation *γ*_*i*_, i.e., *α*_*i*_ = {*α*_1_ × *α*_2_ × *α*_3_ × ... × *α*_*N*_} is factored across each agent at every situation *γ*_*i*_. That is the set of actions given a particular situation. For example, if the situation, *γ*_*i*_, is fire spotted by a UAV, then action, *α*_*i*_, could be making shorter waypoints to map the fire’s shape.
• *λ* is the agents’ waypoint assignment function given the agent’s situation (*γ*_*i*_) and action (*α*_*i*_), such that *λ* : *W* × *γ*_*i*_ × *α*_*i*_ → *A*_*i*,*j*_.
• *P* = {*P*_*A*_, *P*_*M*_, *C*_*o*_} is the set of agent parameters (*P*_*A*_) and mission parameters (*P*_*m*_) (Table 1), and their target cost optimization function (i.e., minimize or maximize from Table 1).
• *δ* is the agent’s probability of changing action in response to the situation. Thus, the probability distribution of *δ* consists of situation action transition values, i.e., *δ* → *α*_*i*_ × *γ*_*i*_. The value of *δ* is initialized using *δ* = 100%/*n*, where *n* is the number of possible situations (*γ*_*i*_) of the sensor states, e.g., fire present or absent. The update (increments/decrements) of *δ*_*i*_ occurs after every sensor poll.
• *S* is the bounded search space segmented into equal cells *s*_*i*_, such that *S*_*i*_ = {*s*_1_, *s*_2_, *s*_3_, …, *s*_*n*_}.
• *S*_*condition*_ is the set of environmental variables that determine the dynamic feature of the target at a given situation and time described by the tuple *S*_*condition*_ = {*γ*_*i*_, *S*_*v*_, *t*_*i*_}, where *S*_*v*_ is the set of environment’s dynamic variables (wind speed, wind direction, fuel type, fuel condition, and terrain nature). The essence of the environmental variables is to describe the changing nature of the operating environment (environmental dynamism).
• *K*_*i*_ is the set of constraints *K*_*i*_ = {*k*_1_, *k*_2_, *k*_3_, …, *k*_*n*_} imposed on the agents, e.g., limited agents energy, limited sensor range, unknown targets locations, and limited communication range.
• I is the agent’s interactions (data exchange through communication based on the agents’ proximity).
• C is a real-valued cost function defined by $C:\lambda_{i}\to R^{+}$ . That is, every agent’s waypoint selection in a particular situation is mapped to a positive real number cost value measured using Table 3.
• *O* = {*o*_1_, *o*_2_, *o*_3_, …, *o*_*N*_} is the set of detecting targets. The targets are moving subjected to the environment condition, i.e., the location of the target at a given time is defined by *O*_*i*_ × *S*_*i*_ → *S*_*condition*_ × *S*_*v*_.

The goal of this study is to develop a MAP/DULAR algorithm that efficiently utilizes the resources in Table 1. That is, to find an efficient set of waypoints plans *π*′ such that $U_{\mathrm{best}}^{\pi^{\prime }}$ is best for every agent situation *γ*_*i*_, i.e., $\pi^{\prime }\in \Pi^{i}U_{\mathrm{best}}^{\pi_{i}}$ where *π*′ = {*π*_1_, *π*_2_, *π*_3_, …, *π*_*i*_} and each of the *π*_*i*_ is the set of that optimize agents’ and mission’s parameters at every situation. U is the best utility consumption function for the set of parameters cost values defined by Eq. 2. The best utility consumption of MAP/DULAR is the set of cost implications at every agent waypoint assignment, i.e., *∀ λ*_*i*_ → *w*_*i*_. The best utility function *U*_*best*_ is described using Eq. 2.

$$U_{\mathrm{best}}\left(C,\lambda ,P\right)={\left[\overset{T=n}{\underset{T=0}{\sum }}\overset{i=n}{\underset{i=0}{\sum }}\left(\vec{C_{i}}\left(\lambda_{i}\backslash P_{i}\right)\right)\right]}^{T},$$ (2)

where $\vec{C_{i}}$ is the agent’s set of cost functions and target optimization vectors at every situation *γ*_*i*_ (as defined in Table 1). In other words, *U*_*best*_ is the function that gives the best utility cost of every parameter (of Table 1) in any situation given a particular waypoint assignment.

## 2 Related Work

Multiagent search continues to be an interest to the AI and robotics communities (Cabreira et al., 2019; Rathbun et al., 2002). The field of multiagent search evolved from the simultaneous localization and mapping (SLAM) area, which involves tasking robots to explore an area and adapt to the elements of the area, e.g., chairs within a room during robots automated vacuum cleaning. (Bonetto et al., 2021; Dissanayake et al., 2001; Kim and Eustice, 2015). Work has focused on computing fixed paths (fixed geometric patterns, e.g., parallel track, creeping line, etc.) for each agent, enabling agents to compute and adapt their paths to the environment, or providing agents with a combination of fixed and adaptive protocols. The fixed-pattern methods follow predefined geometric paths (Cabreira et al., 2018), e.g., expanding squares or parallel tracks to explore the search area (Bevacqua et al., 2015; Jensen-Nau et al., 2021; Huang, 2001; Koenig and Liu, 2001). Related methods such as sector search define angles and edges to control the agents’ paths (Bevacqua et al., 2015; Jensen-Nau et al., 2021). These methods make it easy to compute paths for each agent but do not support adaptation to changes or failures (e.g., sensor or motor failure) in complex and dynamic domains. They struggle in optimizing resource usage or enabling proper coordination between multiple agents (Cabreira et al., 2018, 2019; Di Franco and Buttazzo, 2016).

Grid-based methods that segment the search space into cells impose structure on the problem by constraining random walks (Hackney and Clayton, 2015). The paths followed by each agent and the frequency with which an agent visits a particular cell are controlled probabilistically, e.g., using computational models inspired by the ant pheromone (Cabreira et al., 2019; Di Franco and Buttazzo, 2016; Koenig and Liu, 2001; Nasirian et al., 2021; Zhou et al., 2020). The limitations of such methods include huge computational demands during optimal solutions search and agents’ coordination, e.g., agents’ number of cell (location) visits need to be shared among agents for better coordination (Demyen, 2006; Koenig and Liu, 2001).

There is also work inspired by animal foraging with random waypoints within the search space (Chawla and Duhan, 2018; Sutantyo et al., 2011). The key advantage of such pseudorandom methods is the agents’ independent planning, which supports decentralized coordination. At the same time, these methods can suffer from poor agent coordination in complex domains, difficulty in predicting the future activities of the agents, and limited consideration of the agents’ sensing abilities (Nurzaman et al., 2009; Cabreira et al., 2019).

The aim of hybrid approaches is to combine the strength of fixed paths and pseudorandom methods using agents control protocols. The works of Sutantyo et al. (2011) and Chawla and Duhan (2018) describe various hybrid approaches to pseudorandom methods in which local agents protocols are applied. The most common protocol is to generate waypoint pseudorandomly and prioritize areas with the most targets detection as inspired by artificial potential field, ant colony optimization, and bat algorithms. (Chawla and Duhan, 2018; Sutantyo et al., 2011). There are other forms of hybrid methods that use geometric processes, e.g., Voronoi tessellation in pure form or augmented with order processes, e.g., buffering (Arul and Manocha, 2021), k-means algorithm (Chowdhury and De, 2021), gradient descent algorithm (Inoue et al., 2021), area prioritization (Zarei and Mozafar, 2021), and particle swarm optimization (Zaimen et al., 2021). In these methods, the plan generation is controlled by theorems, propositions, lemmas, and protocols of the geometric process. These hybrid approaches resemble the proposed method of the Delaunay-inspired approach (presented in Section 3), with the main limitation of focusing on local agents rules rather than both local and system protocols. In the Delaunay-inspired method, waypoints are generated from the centers of the triangles rather than the centers of the circumcircles as adapted by Voronoi methods. Our characterization of the multiagent search problem considers resources (Table 1) utilization, scalability, adaptability, and predictability as the primary key features of concern. Thus, we present a hybrid approach that combines the strengths of fixed-path and pseudorandom methods to address the outlined limitations.

## 3 The Proposed Solution

**FIGURE 2:**
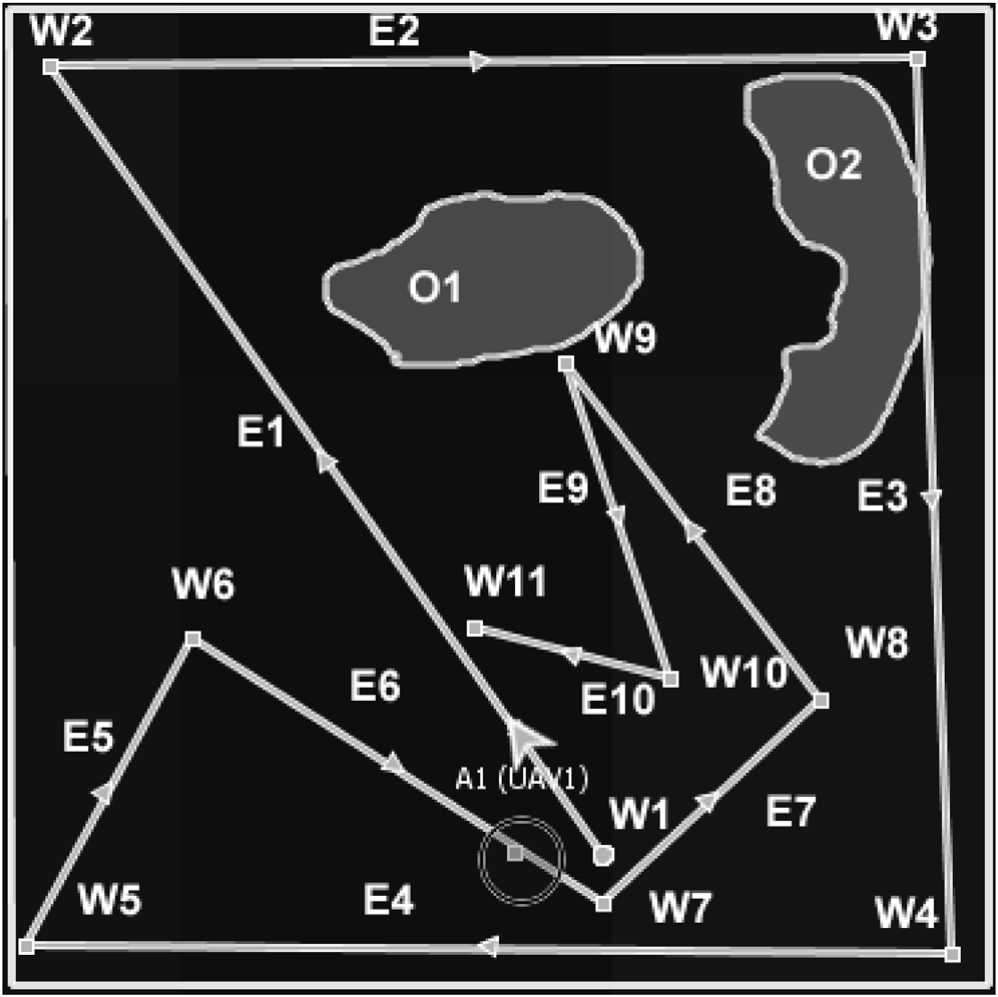
Example of the Delaunay-based solution for one agent (UAV1). Agents paths can be traced using the directional arrows.

**FIGURE 3:**
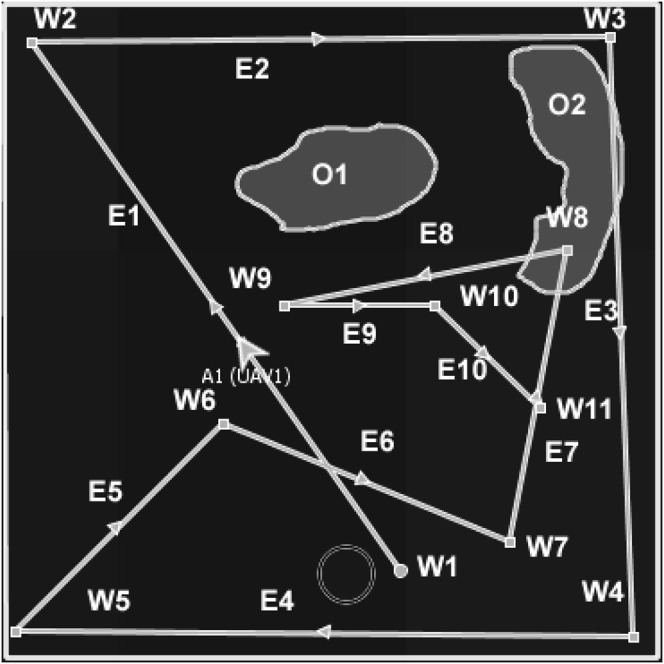
Example of the DIMASS MAP/DULAR solution for one of the UAVs (UAV1).

The proposed algorithm evolved by applying a Delaunay triangulation to seed waypoints (seed waypoints are defined heuristically, i.e., using structured rules, e.g., the longest non-crossing paths from the agent’s current location as described in Figures 2 and 3; or derived from a known predictable distribution), through the inscription of triangles around each waypoint in a circle to avoid overlap of waypoints (Cignoni et al., 1998). The initial version of the algorithm (in Appendix 1) systematically generates seed waypoints for the first layer waypoints (by taking the longest non-crossed paths from the agent’s current waypoint) and then performs Delaunay triangulation of the seed waypoints. The centre of each triangle is a planned waypoint, and the process is repeated until the number of waypoints is less than or equal to 2 (at this stage, triangulation is not possible because it requires at least three waypoints). Each set of the Delaunay triangles makes a MAP/DULAR layer (Definition 3.1). The number of triangles and edges of the Delaunay triangulation are computed as 2*n* − 2 − *k* and 3*n* − 3 − *k*, respectively, where *n* is the total number of waypoints and k is the number of convex hull waypoints (Perera and Barnes, 2011), i.e., a theorem for computing the number of triangles and edges. Figure 2 describes the implementation of this version of the algorithm. From Figure 2, waypoints labeled *W*1 to *W*5 are the longest non-crossed paths from the UAV’s (UAV1) current location. Waypoints labeled *W*6 to *W*9 are the centres of the Delaunay triangles of waypoints *W*1 to *W*5, which make the layer 2 waypoints (see Definition 3.1). Similarly, waypoints *W*11 and *W*12 are the centres of the Delaunay triangles of waypoints *W*6 to *W*9, which made the final layer (layer 3) waypoints. This is similar to the Voronoi tessellation methods (Arul and Manocha, 2021; Chowdhury and De, 2021; Inoue et al., 2021) in which waypoints are the centers of the Delaunay triangulation circumcircles instead of the centers of the triangles. The AMASE simulator calls the Delaunay triangulation methods and generates the waypoints. The visualization of the Delaunay triangulation of the waypoints is described in supplemental documents.

Definition 3.1 [MAP/DULAR Layer] MAP/DULAR layer (*τ*_*i*_) refers to the set of waypoints at the same level of the plan, i.e., *τ*_*i*_: *W*_*x*_ → *A*_*ij*_, such that *W*_*x*_ = {*w*_1_, *w*_2_, *w*_3_, …, *w*_*m*_}, *∀ W*_*x*_ ∈ *W* and *∃ τ*_*j*_ = *W*_*y*_ → *A*_*ij*_, where *W*_*y*_ = {*w*_1_, *w*_2_, *w*_3_, …, *w*_*n*_}, such that *W*_*x*_ ∩ *W*_*y*_ = {} *∀ W*_*x*_, *W*_*y*_ ∈ *W*. Waypoints in every layer are characterized by edge length, quadrants, and projection angles.

Algorithm A1 of Appendix 1 shows the initial version of the algorithm, and Figure 2 describes a solution generated by one of the UAVs while solving the problem in Figure 1. The seed waypoints labeled *W*1, *W*2, *W*3, *W*4, and *W*5 are the longest non-crossed paths based on the agent’s current location, which serve as the seed waypoints (first layer waypoints). The second layer waypoints are the waypoints *W*6, *W*7, *W*8, and *W*9, which are the centres of the Delaunay triangles of the first layer’s waypoints. Layer 3 waypoints are *W*10 and *W*11, which come from layer 2 triangles’ centres. The outcome looks predictable because agents’ future waypoints can be estimated if the initial waypoint, speed, and waypoints generation rules are known.

In addition, the outcome has highly spread waypoints (by giving highly spread waypoints across the search space) and can be partly controlled (by changing the seed waypoints or waypoint generation rules). For multiple agents, the seed waypoints can be varied, e.g., using Definitions 3.2 and 3.3, and unique searching waypoints would be obtained because of the Delaunay triangulation unique waypoints generation when given varying sets of seed waypoints (Cignoni et al., 1998; Demyen, 2006; Kallmann, 2005). Further refinement of the algorithm produces an approach we call DIMASS (Delaunay-Inspired Multi-agent Search Strategy), as described in Algorithm 1. This is a simplified, efficient, predicable, adaptable, and scalable version of the above-described Delaunay-based solution (based on the comparison of the results in Section 5 and the initial version of the algorithm from Appendix 1).

Algorithm 1 DIMASS: Delaunay-Inspired Multi-Agent Search Strategy.

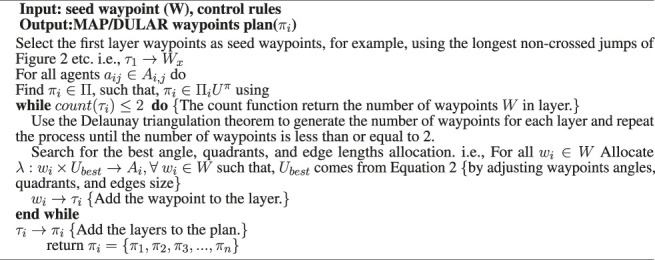

In DIMASS (Figure 3), when an agent obtains seed waypoints W1 to W5 of Figure 2, rather than performing triangulation, it uses navigation rules based on the Delaunay triangulation theorems to generate waypoints in the upper layer. For example, the waypoints W6, W7, W8, and W9 of Figure 2 were obtained by projecting in angle = 180°/*n*, where *n* is the number of upper-layer waypoints computed using the Delaunay triangulation process. The projection quadrants use different sequences depending on the number of agents and paths needed. For instance, UAV 1 could use the first, third, second, and fourth quadrant sequences, while other UAVs could use the third, fourth, second, and first quadrants. That is, using different waypoints edges (*E*_*i*_) for *i* ∈ [1, *N*], projection angles, and quadrants can be customized to effectively utilize resources (by avoiding redundant search and maximizing coverage). For example, the edges of the second layer waypoints (*W*6 − *W*9) from Figure 3 are half of the opposing edge (i.e., *E*5 = *E*4/2), etc. Searching the best combination of angles, quadrants, and edges for each agent is computationally cheap because the highest number of quadrants is only four, while edges and angles can be controlled by setting a range of values, e.g., ranges of 24° for angle difference (i.e., 360°/15, which means 15 number of searches). Therefore, Figure 3 is only one of the possible solutions. Thus, the best solution has the best configuration of projection angles, quadrants, and edges selection to solve the problem in Eq. 2.

Having defined a set of paths, the next challenge is to ensure that these paths satisfy the resource constraints outlined in Table 1, i.e., the DCOP solution of Eq. 1, which is based on adjusting waypoints edges, angles, and quadrants until resources consumption is efficient given the agents’ current situations and time limits (finite-horizon). For the purpose of layers selection and waypoints allocation, the following concepts are important.

Definition 3.2 (Reflected Waypoints). Two waypoints *X*_*ij*_ and *Y*_*ij*_ with dimensions *i* and *j* and search space lower and upper boundaries *M*_*ij*_ and *N*_*ij*_, where $i,j\in R^{d}$ , within a MAP/DULAR plan of d-dimension (note that, for i and j, d = 2), are said to be reflected if and only if the distance computation in Eq. 3 exist.

$$Y_{i}=N_{i}-\left(X_{i}-M_{i}\right)\;or\;Y_{j}=N_{j}-\left(X_{j}-N_{j}\right),$$ (3)

where $i,j\in R^{d}$ .

Definition 3.3 (Refracted Waypoints).

Two waypoints *X*_*ij*_ and *Y*_*ij*_ in a MAP/DULAR environment with search space lower and upper boundaries *M*_*ij*_, *N*_*ij*_, where $i,j\in R^{d}$ are said to be refracted waypoints if and only if the distance computation in Eq. 4 exist.

$$Y_{ij}=N_{ij}-\left(X_{ij}-M_{ij}\right),\;\;i,j\in R^{d}.$$ (4)

**FIGURE 4:**
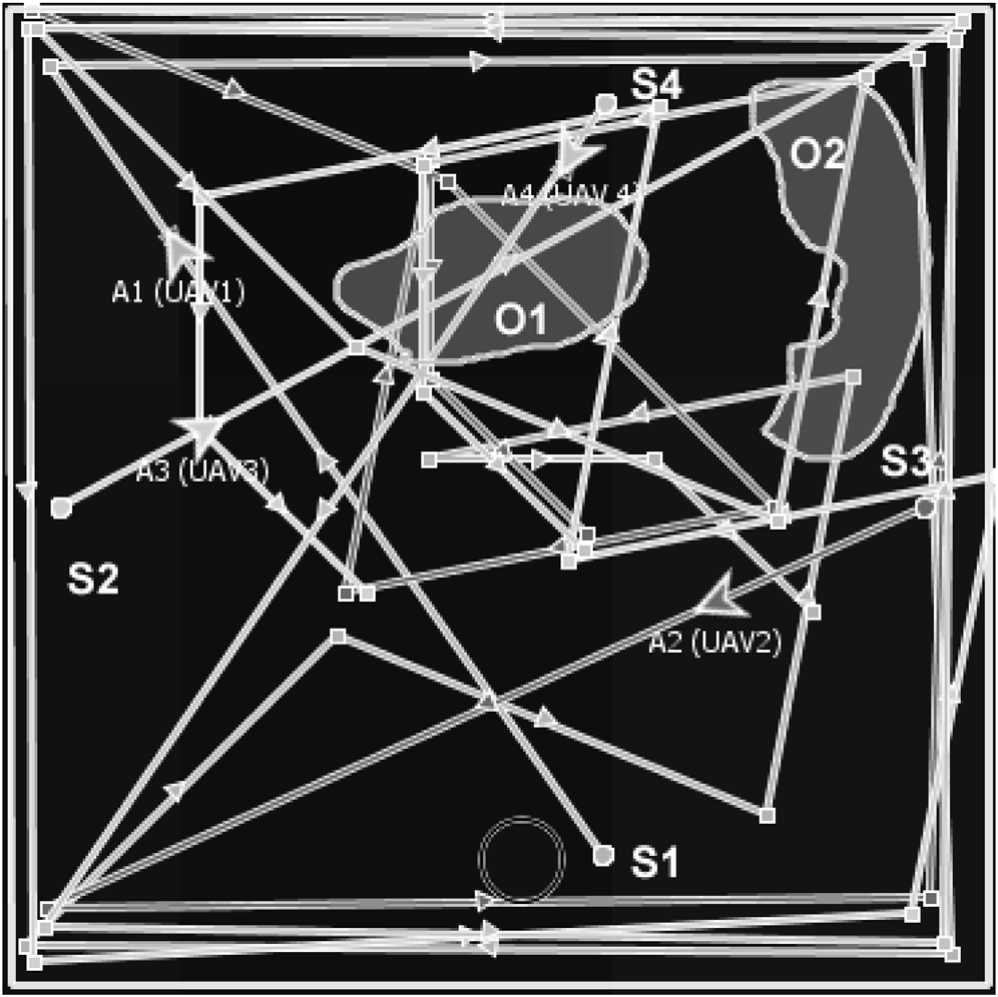
The MAP/DULAR solution for a team of four UAVs.

Reflected or refracted waypoints resemble light rays and can coordinate multiple agents. For example, Figure 4 describes the MAP/DULAR solution for four UAVs using the DIMASS algorithm. UAVs A1 and A4 have refracted seed waypoints starting from *s*1 and *s*4, and agents A2 and A3 have reflected seed waypoints starting from *s*2 and *s*3. Paths can be traced by following the directional arrows. Each of the UAVs from Figure 4 is on its path. Thus, each individual agent has a unique path. Note that the overlapping paths from Figure 4 will be explored at different times by different UAVs based on the concept of waypoints reflection and refraction (as originated from the initial location and seed waypoints differences). As such, the paths will not be considered redundant due to exploration time differences. Thus, two different UAVs with distinct seed waypoints and/or waypoints generation protocols have distinct MAP/DULAR solutions using DIMASS based on the uniqueness of the inspired Delaunay triangulation (Cignoni et al., 1998). Similarly, Proposition 1 proved that the convex plans (plans with waypoints interior angles less than 180°) have higher path divergence (areas of exploration spread across the search space as defined in Section 5) than the concave ones.

Proposition 1 (path divergence of MAP/DULAR problem). *Convex MAP/DULAR has more path divergence than the concave counterpart with the same edges configuration.*

Proof: Let *n* be the number of waypoints, and the convex of n be *Conv*(*n*). If m out of n waypoints of a concave plan are concave, then the convex hull of Δ_*convex*_ of the concave plan is *Conv* (*n* − *m*), i.e., using Graham’s Scan algorithm. From Euler’s formula, the number of triangles formed for the convex plan Δ_*convex*_ is Δ_*convex*_ = 2 + *E* − *n*, while for the concave plan is Δ_*concave*_ = 2 + *E* − (*n* − *m*). Therefore, the total area of the triangles formed is $\sum_{i=1}^{n=n}\Delta_{\mathrm{convex}}>\sum_{i=1}^{n=n}\Delta_{\mathrm{concave}}$ because *n* > *n* − *m* for *m* > 0 and the edges are of the same size.

## 4 Evaluation

The metrics used for the evaluation are qualitative and quantitative. The quantitative metrics are coverage, path divergence, energy, mission time, computational power (measured by time complexity and McCabe cyclomatic complexity (McCabe, 1976)), memory use, and redundant search. The qualitative metrics (non-functional features) are scalability, predictability, and adaptability. The metrics can be used in determining the mission utility cost and the overall efficiency of the algorithms.

**FIGURE 5:**
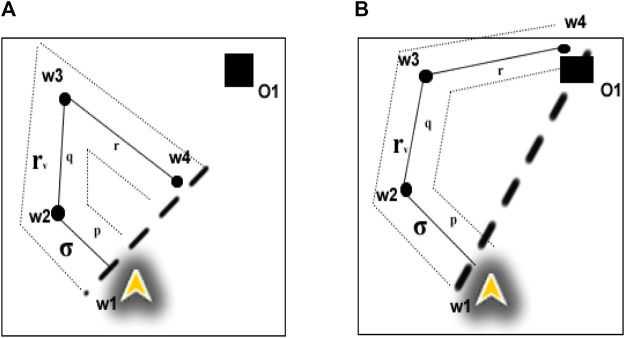
Example of MAP/DULAR path divergence variation for two plans.

Coverage is the proportion of search area with sensed agents path, i.e., $\sum_{i=1}^{i=n}S_{i}$ such that *∀S*_*i*_ *∃w*_*i*_ × *r*_*v*_ ∈ *S*_*i*_, where *r*_*v*_ is the sensing range. In other words, coverage is the portion of the search space S with the agent’s path and sensing. This can be measured by segmenting the search space into cells of equal sizes (*S* = {*s*_1_, *s*_2_, *s*_3_, …, *s*_*n*_}) and counting the cells with paths and sensing in them (Sauter et al., 2005; Sutantyo et al., 2011). One of the key limitations of measuring coverage in this way is that cells with paths close to them could have a partial visit, i.e., $\sum_{i=1}^{i=n}S_{i}$ such that *∀S*_*i*_ *∃w*∉*S*_*i*_ and *r*_*v*_ ∈ *s*_*i*_, and, *∃* distance *d* = ‖*s*_*i*_ − *s*_*j*_‖ such that there exists one of the following conditions: *w*_*i*_∉*S*_*i*_ and *r*_*v*_ ∈ *s*_*i*_ or *w*_*i*_ ∈ *S*_*i*_ and *r*_*v*_∉*s*_*i*_ and the area of *d* < *s*_*c*_ where *s*_*c*_ is cells’ size, and not counted as covered. In other words, partially visited cells have no agents path (waypoint path) in them but have partial sensing (*r*_*v*_) due to their proximity to the path. Thus, because of the absence of a path, they will not be counted as covered. Considering the omission of partial visit limitation, we then introduce the path divergence metric, which is the measure of how the search path is spread across the search space, i.e., by considering waypoints spread within the search space. For example, the plan in Figure 5 picture “B” has higher path divergence than the one in picture “A” because its paths have higher separation across the search space. The plan in picture “A” can be stretched to have more path divergence simply by changing the angles, quadrants, and edge length configurations. We measure the path divergence by summing up the area of the Delaunay triangles of the waypoints, i.e., *∑area* (*Delaunay* (*W*_*i*_)), the area function compute the area of the Delaunay triangles of the set of waypoints *W*_*i*_. In addition, path divergence can be used in controlling redundant search (i.e., continuous exploration of certain locations many times within a short period).

Energy is the amount of power consumed when performing mission activities. We compute energy consumption by taking the percentage of battery consumed by a real UAV given certain flight modes (ascending and descending). The DJI Ryze Tello Edu drone was used to obtain the agents’ energy consumption. The drone was tasked to perform different flight modes using the Tello Python application programming interface (API)^3^ for the range of 500 cm, and the battery level in percentage was monitored. Note that the energy consumption rate depends on the UAV type and operating environment condition (*S*_*condition*_ defined in Section 1.1), e.g., consumption of 0.049%/s during UAV ascending. The evaluation process considers different flight modes (as described in the results in Table 5) and assumes constant sensing as adapted from Huang (2001). Energy is important due to the limited battery capacity of the UAVs (Cabreira et al., 2018, 2019; Merino et al., 2006, 2010; Jensen-Nau et al., 2021). Mission time is the amount of time spent while conducting the mission. This is measured simply by using the mission clock of the AMASE simulation. Mission time relates to coverage and other parameters. For example, an algorithm that covers an area of 100 km square (100* *km^2^) in 1 min is more effective in terms of mission time than an algorithm that covers the same area in 5 min.

Time complexity is the measure of the computational power needed to execute the algorithm. This metric is essential in determining the capacity of the agent to be used, i.e., complex algorithms require agents with larger computational capacities. In addition, McCabe cyclomatic complexity (McCabe, 1976), which measures the number of branches (a branch could be a loop or a function) for the algorithm implemented, was used to measure the algorithms’ implementational measures. McCabe’s cyclomatic complexity is similar to the time complexity, i.e., an algorithm with low cyclomatic complexity could be tested, evaluated, and implemented more easily than one with a higher number (McCabe, 1976; Wallace et al., 1996). Eclipse cyclomatic complexity plugin was used for the cyclomatic complexity measurement.

Redundant search is the number of times a particular space is explored within a short period of time. This is measured by counting the number of consecutive waypoints generated within a sensor range interval (i.e., the overlapping sensor range for two subsequent waypoints). Redundant search is categorized into intra-agent and inter-agent redundancies. Intra-agent redundancy refers to the overlapping waypoints among the agent’s self waypoint, whereas inter-agent redundancy is the overlapping waypoints among two different waypoints. These quantitative parameters are related to one another, as implied in Table 1, and depend on mission requirements and agents’ capacity. For example, coverage is critical during searching and less critical during mapping. Similarly, running computationally expensive algorithms on micro UAVs can be critical.

The benchmark values for the metrics are in line with the reported values from the literature. For example, agents’ searching mission time can be reduced by 50% when the Lévy flight (a popular pseudorandom search) algorithm (Chawla and Duhan, 2018; Sutantyo et al., 2011) is augmented with artificial potential fields instead of being purely pseudorandom (Sutantyo et al., 2011). Similarly, the work of (Sauter et al., 2005) shows that agents’ search coverage of fixed-pattern methods has a 25% chance of having higher coverage at the first 80 min of the agent’s mission than a fixed-pattern method augmented with pheromone inspiration. The rest of the metrics (with the exception of path divergence, McCabe cyclomatic complexity, predictability, and adaptability as introduced in this paper) have been reported in (Huang, 2001; Jensen-Nau et al., 2021; Kanistras et al., 2013; Koenig and Liu, 2001; Li et al., 2011; Sutantyo et al., 2011). Therefore, while we are reporting our specific results, we acknowledged the standards and similar patterns presented by existing works in line with our outlined contributions.

The qualitative metrics are non-functional features (predictability, scalability, and adaptability) of the algorithms. Predictability is a measure of how the algorithms allow agents’ location predictions (location estimation based on known parameters, e.g., speed, downwind and upwind acceleration and retardation, etc.). For example, due to the randomness of the Lévy flight, each round of waypoints plan generation is different (Chawla and Duhan, 2018; Sutantyo et al., 2011; Yoon and Kim, 2013) which makes prediction impossible or very difficult. Predictability can be measured using the standard deviation of the number of generated waypoints by an algorithm given the same time and structure constraints. In order words, predictability can be measured as the function of the structure of the generated plan. For example, DIMASS and fixed-pattern methods stick to a stable number of waypoints when seed waypoints and track configurations are the same. Considering the plan generated in Figure 3, each agent has the same number of waypoints (i.e., a total number of 11 waypoints based on the five seed waypoints) and similar edges, quadrant, and angle configuration patterns. This means the plan is structured, unlike the pseudorandom methods that generate a varying number of waypoints (based on the standard deviation in Tables 7 and 8). That is, predictability leads to a uniform standard deviation in the number of generated waypoints across the agent’s generated plan. For example, considering the DIMASS plan in Figure 3, the standard deviation of the number of waypoints will always be $i\sum_{i=0}^{i=n}\left(p_{i}-q_{i}\right)$ , where *i* is the number of plans generated, *p* is the number of generated waypoints, and *q* is the mean of the number of waypoints generated at each plan *π*_*i*_. Thus, for Figure 3, as far as the plan seed waypoints and edges, angles, and quadrants generation rules are the same, the standard deviation will be 0, i.e., *i* (11 − 11) across all layers for Figures 3 and 4. On the other hand, pseudorandom methods generate a different set of waypoints plan at each iteration of the algorithm with a varying number of unpredictable waypoints based on the standard deviations as described in Tables 7 and 8. Adaptability is a measure of how the algorithms can be customized to suit certain functionalities, e.g., directing the final waypoints near a charging point, narrow space exploration, etc. We measured adaptability by counting the number of controllable path elements, i.e., the path’s quadrants, angles, and edges. For example, DIMASS can be controlled by changing edge, quadrants, and angle configurations; as such, the adaptability number is 3 (Table 10). The pseudorandom method, e.g., the Lévy flight, cannot be controlled even if the seeds of the random numbers are clustered between ranges (as discussed in Section 5). Summarily, adaptability is the feature of the algorithm that allows it to be applied for more than one purpose, e.g., area coverage search, mapping, etc. The scalability of an area coverage planning algorithm is the measure of its ability to handle multiple numbers of agents with resource stability. This is measured using both the time and implementational complexity of the algorithms. For example, assume two algorithms A and B; algorithm A is more scalable than B if it can handle a higher number of agents with stable time or cyclomatic complexity than B. Overall, the higher the number of quantitative and qualitative metrics utilized by a MAP search (area coverage algorithm), the better.

### 4.1 Experiment Design

**FIGURE 6:**
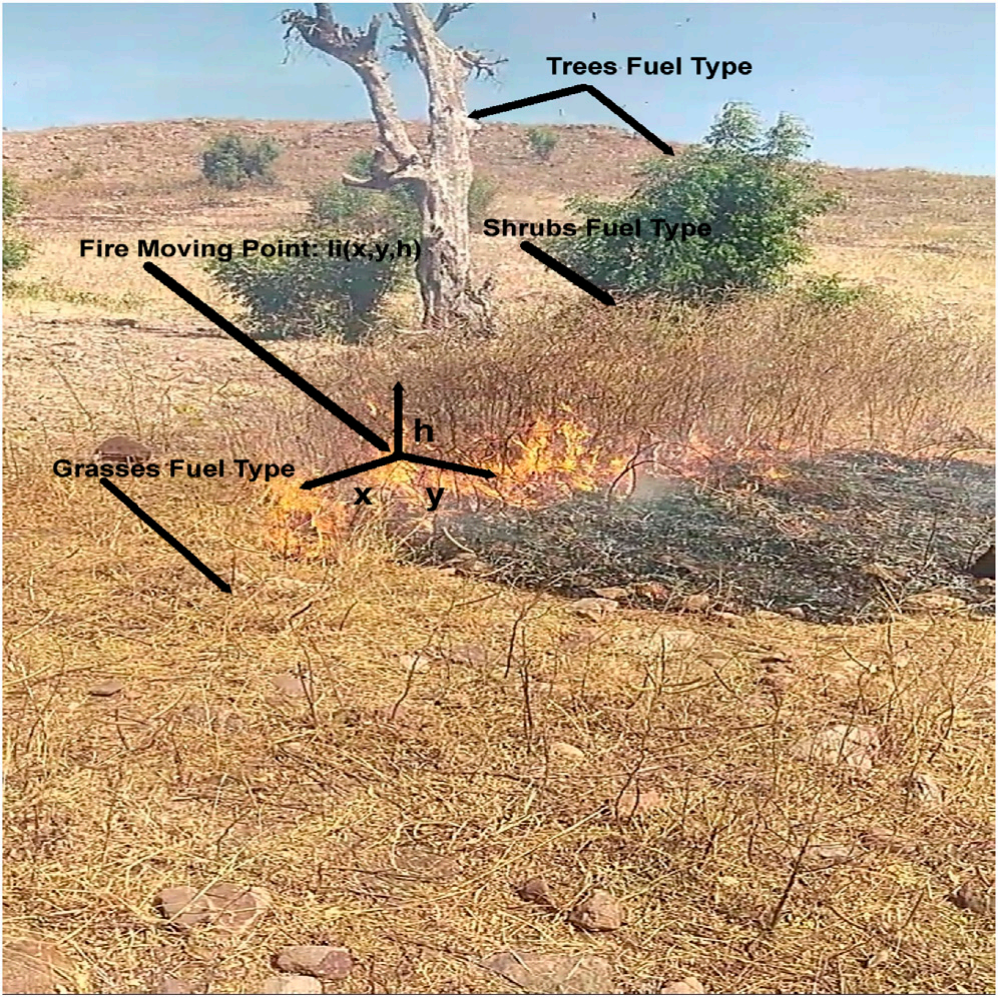
Fire spread rate measurement experiment.

**FIGURE 7:**
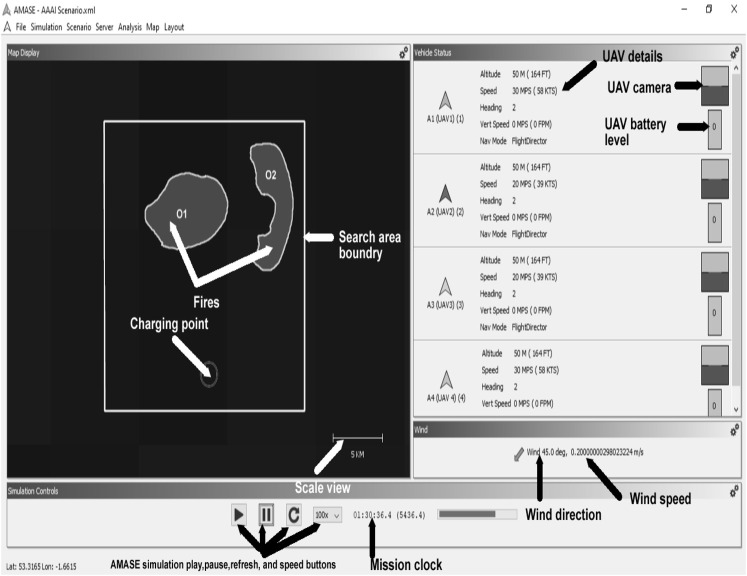
AMASE experiment running with fires moving (mission clock 01:30).

The experiment employs an AMASE model of the problem described in Figure 1. Targets (fires), fuel types, and other dynamic variables (wind speed, wind direction, fog, and clouds) were simulated. For example, the growth rate of the fire at every location $l_{i}{\left(x,y,h\right)}^{t_{i}}$ in time difference Δ*t* = *t*_*i*+1_ − *t*_*i*_ is defined using Eq. 5

$$l_{i}{\left(x,y,h\right)}^{t_{i+1}}=l_{i}{\left(x,y,h\right)}^{t_{i}}\times w^{t_{i}}\left(\Delta t,S_{v}\left(\vec{x}\right),S_{v}\left(\vec{y}\right),S_{v}\left(\vec{h}\right)\right).$$ (5)

where ${\left(x,y,h\right)}_{i}^{t}+1$ is the estimated growth of the target (fires) across *x*, *y*, *h* axis (where *h* is the height) from the current position ${\left(x,y,h\right)}^{t_{i}}$ , Δ*t* is the time interval, and $w^{t_{i}}\left(\Delta t,S_{v}\left(\vec{x}\right),S_{v}\left(\vec{y}\right),S_{v}\left(\vec{h}\right)\right)$ is the function that defines the growth factor by considering the current environmental dynamic variables, (*S*_*v*_), factors (wind speed, wind direction, and fuel type) and time interval Δ*t*. That is, each of the functions $S_{v}\left(\vec{x}\right),S_{v}\left(\vec{y}\right),S_{v}\left(\vec{h}\right)$ returns the velocity vector of the target (fire) mobility rate for each dimension based on the environmental dynamic variables configuration vector taken as passing parameter, e.g., fuel type, wind speed, location relation ground surface (uphill or downhill, etc). Parameters values for the variables were obtained from documented standard operating procedure (SOP), UAV images analysis works (Fernández-Hernandez et al., 2015; Haddadi et al., 2020; Dalla Corte et al., 2020; Corte et al., 2020; da Costa et al., 2021; Mohan et al., 2021; Neto et al., 2021), and arranged physical experiments as described in Figure 6 and Table 4. For example, the contribution to fire spread weight (w) from dried shrubs is higher than the wet ones (fire spreads faster in dried shrubs than in marshland). Similar weighting is performed for the influences of other variables, such as wind speed, wind direction, and location relation to the ground. The higher the weight (w of Eq. 5), the higher the translation (growth) rate. Other dynamic variables such as wind speed or wind direction follow defined dynamic generation patterns. For example, the wind speed could be changing values systematically every time interval Δ*t* (as shown in Figure 7). Fuel type and location relation to ground (uphill, downhill, or flat) will be defined based on the location values using search area segmentation *S*_*i*_ (by dividing the search space into cells of equal sizes). All these variables have their corresponding element definition tag in XML of the AMASE view. Alternatively, dynamic variable features can be inserted using backend manipulations (using the provided AMASE Java library). The UAVs’ features and sensor configurations are defined in a similar way. For example, Table 5 describes the UAVs’ variables and sensor configuration. The issues of sensor reliability and conflict were beyond the scope of this study; as such, the reader is referred to our previous work (Yusuf and Baber, 2020, 2022) for more details on sensor issues. The UAVs’ energy consumption across various flight modes for the AMASE simulation utilizes the real UAVs experiment derived values.

**TABLE 4:** Fire spread values.

Location ID *#*	Spread length (Meter per second)	Experiment time (minute)	Direction of spread
1	0.002	14	East
1	0.009	14	Northeast
1	0.005	14	West
1	0.006	14	North
2	0.004	8	Southeast
2	0.025	8	Northwest
2	0.02	8	Southwest
2	0.0135	8	South

**TABLE 5:** UAVs simulation details.

UAV type	Flight type	Speed (m/s)	Vertical speed (m/s)	Pitch angle (*x*°)	Max. Bank angle (*x*°)	Min./Max. Speed (m/s)	Energy rate (%/second)
Fixed-wing	Cruising	30	0	0	30	10/40	0.049
	Loitering	20	0	5	30	10/40	0.0083
	Ascending	30	5	10	30	10/40	0.05
	Descending	30	−5	−5	30	10/40	0.025
	Dashing	40	0	−2	10/40	0.019	
Multi-copter	Cruising	20	0	0	30	0/25	0.074
	Loitering	20	0	5	30	0/25	0.037
	Ascending	20	5	10	30	0/25	0.075
	Descending	20	−5	−5	30	0/25	0.05
	Dashing	25	0	−2	30	0/25	0.049

The target (fire) spread values across different environmental conditions (e.g., various values of wind speed) were obtained from a physical experiment. The experiment was conducted in Nigeria (i.e., the fire spread values collection experiment) within a government-approved quarrying area at the beginning of a dry season on top of a hill. The plants on top of the hill are set on fire intentionally at the beginning of every dry season to clear the shrubs, trees, and grasses for easy access. (That is why some of the trees are dead in Figure 6). This traditional clearance method was used during the fire spread values collection (results reported in Table 4). The hill is surrounded by a trench to avoid fire escalation. All permissions were granted, and all necessary safety measures were observed before starting the fire. The location has a combination of dried and semi-dried trees, shrubs, and grasses, as shown in Figure 6. The observations were made on a sunny day with temperature, wind speed, and humidity values of 34°C, 8Kilometer per hour, and 12%, respectively. The fire spread rate was measured by making marks across the directions (north = 0°, east = 90°, south = 180°, west = 270°, northeast >0° and <90° , southeast >90° and <180° , southwest >180° and <270° , and northwest >270° and <0°(360°) , as defined by the American Practical Navigator (Bowditch, 2002).

Table 4 describes the excerpt of the fire spread values across two locations in varying directions and time frames (target height was measured relatively the same as fuel height), and the full parameters were available in the simulation XML source code inside the supplemental documents folder. The spread rate value depends on the location terrain nature (Ingle, 2011; Merino et al., 2006, 2010), fuel, e.g., trees, shrubs, grasses, etc., as described in Figure 6, environmental condition (*S*_*condition*_), and other dynamic variables, e.g., wind speed, wind direction, etc. Figure 7 describes the AMASE simulation of the problem at 1:30:36.4 mission clock, i.e., the figure shows fire expansion and other simulated variables, e.g., wind speed, wind direction, UAV battery etc. values. Thus, the simulated environment presents a dynamic environment. Tables 5 and 6 describe the UAVs and sensors configurations based on the DJI Phantom 3 functionalities and capacities.

**TABLE 6:** Target detection sensor configuration.

Sensor type	Video stream X/Y resolution (px)	Min. X/Y view (m)	Supported wavelength band	Elevation	Target detection range (Microns)
Infrared Camera Type 1	256/192	55/55	Short-wave infrared	450	0.9–1.7 microns
Infrared Camera Type 2	256/192	55/55	Mid-wave infrared	450	2–5 microns
Infrared Camera 3	256/192	55/55	Mid-wave infrared	450	7–12 microns
Spectrum Camera	256/192	55/55	Electro-optical	450	0.4–0.76 microns
Thermistor (Temperature Sensor)	-	-	Heat sensor	450	87^*o*^

## 5 Results

The simulation experiment was designed to test the hypothesis that the proposed solution (DIMASS) is resource-efficient, i.e., it adheres to both quantitative and qualitative metrics utilization in comparison with the existing methods (parallel track, creeping line, expanding squares, sector search, Zamboni, and Lévy flight) based on the described parameters and constraints; and that DIMASS provides a structured search plan which exploits the best features of pseudorandom and fixed-pattern methods. The evaluation results are categorized into agent and mission parameters, algorithm parameters, and qualitative parameters.

### 5.1 Agents and Mission Parameters Performance

Redundant search (both inter-agent and intra-agent redundancy), coverage, and path divergence were evaluated. These parameters are separated because of their strong relation with the agents and mission activities, whereas the next subsection focuses on the algorithms’ computational and implementational analysis.

#### 5.1.1 Redundant Search

Redundant search in fixed-pattern methods can be avoided by configuring the sensor range in line with the inter-track distance (e.g., the distance between the tracks in a parallel track). However, the comparison starts with sensor range, *r*_*v*_ = 5% of the searching space, which make all the waypoints of the parallel track, creeping line, and expanding square methods redundant. Sector search and Zamboni produce a low number of redundant waypoints due to their path spread, as shown in the supplemental document, i.e., no subsequent waypoints overlapping due to their waypoints separation. Therefore, the comparison considers Lévy fight and DIMASS. Because of the randomness of the Lévy flight, mean and standard deviation were calculated from fifteen (15) experiments. The number 15 is to justify the minimum angle used for DIMASS from Figure 3, i.e., 30°. Thus, this is approximately equal to 15 iterations for the DIMASS process of searching for the best angles, edges, and quadrants combination (i.e., 360°/15 = 24°). Table 7 describes the redundant search performance comparison. Each of the entries in Table 7 was obtained by simulating the paths (e.g., Figures 2 and 3) and counting the overlapping consecutive waypoints. For instance, from Figures 2 and 3, the intra-agent redundancy of 2 for DIMASS happens when the sensor range is *r*_*v*_ = 10% and is for the waypoints W10 and W11. The number of redundant waypoints keeps increasing when the sensor range becomes bigger and bigger. For the Lévy flight method, intra-agent redundant waypoints are detected at every subsequent waypoint generation, whereas inter-agent redundancy is detected by exchanging the waypoint information among agents. A total number of 15 rounds of different AMASE missions was used for the Lévy flight, and the mean and standard deviation were reported.

**TABLE 7:** Redundant search performance comparison.

Sensor Range (*r*_*v*_)	D (%)IMASS intra-agent redundancy	DIMASS inter-agent redundancy	Lévy flight intra-agent redundancy	Lévy flight inter-agent redundancy
5	0	1	4 (2)	4.7 (2.11)
10	2	2	9 (4.76)	9.8 (4.80)
15	3	3	7.9 (3.75)	9.8 (4.13)
20	4	4	9.4 (3.37)	10.9 (3.11)
25	4	4	11.2 (2.48)	13.3 (2.54)
30	5	4	11.5 (3.84)	14.2 (4.09)
35	6	4	14.9 (6.89)	18.2 (8.04)
40	6	4	12.6 (3.17)	15.3 (3.89)
45	6	5	17.6 (3.60)	18.26 (2.77)
50	6	7	18 (2.83)	21.6 (3.89)

Table 7 shows that the proposed DIMASS minimizes the number of redundant waypoints (both intra-agent and inter-agent redundancies). The Lévy flight shows more occurrence of redundant search and random behaviour (as can be seen from the standard deviation values, *σ*). The result of DIMASS in Table 7 is one of the possible solutions, and the best solution can be found by stretching the angles, edges, and quadrants to avoid redundant waypoints. Therefore, we can conclude that, although DIMASS demonstrates good performance in terms of redundant search, it is not as good as the Zamboni or sector search. Again, the best DIMASS solution (the DIMASS solution with the best angles, edges, quadrants, and configurations) can provide a zero-tolerance for redundant waypoints whenever possible.

#### 5.1.2 Coverage and Path Divergence

Table 8 shows the coverage and path divergence performance across the selected comparing methods. The coverage is reported as the proportion of the total number of cells. The total number of cells is 10, which is the same as the assumed sensor range for coverage evaluation. As such, the value 1 means full coverage, i.e., 10/10 for all cells covered. For the Lévy flight, the mean and standard deviation of the values of 15 rounds of AMASE missions were reported.

**TABLE 8:** Coverage and path divergence performance comparison.

Algorithm	Path divergence (*km* ^2^)	Coverage
DIMASS	962.33	1
Lévy flight (Chawla and Duhan, 2018)	434.17 (281.20)	0.64 (0.28)
Parallel track (Bevacqua et al., 2015; Jensen-Nau et al., 2021)	128.03	1
Creep lining (Bevacqua et al., 2015; Jensen-Nau et al., 2021)	252.64	1
Sector search (Bevacqua et al., 2015; Cabreira et al., 2019)	588.55	0.69
Expanding squares (Bevacqua et al., 2015; Cabreira et al., 2019)	172.28	0.75
Zamboni search (João, 2012)	518.06	1

From Table 8, DIMASS and some fixed-pattern methods demonstrate high performance in terms of coverage. The path divergence performance varies based on how the search method spreads its path across the search area. For example, considering the stable time and energy allocation, it is obvious that the parallel track and creeping line will have low path divergence based on their structure (as described in their respective pictures from the supplemental documents), especially when the inter-track distance is small. Thus, Table 8 shows that the path divergence and coverage vary based on the path structure. As expected, the Lévy flight demonstrates random behaviour in terms of coverage and path divergence, which is luckily better than some of the fixed-pattern methods. The results show higher performance in DIMASS coverage and path divergence despite not being the best solution of DIMASS (i.e., DIMASS best solution configure the best configurations of angle, edge, and quadrants).

Regarding the relationship between the number of uncovered cells of a DIMASS and coverage plan. The square root of the total number of cells (*S*_*n*_) and the sensor range (*r*_*v*_) were used for the computation. For example, if the number of cells is 100, then the square root (*S*_*n*_) is = 10. An observational result for a single UAV (Figure 3) shows that when (*S*_*n*_) = 4, *C*_*uc*_ = 0 (number of uncovered cells), and when *S*_*n*_ > 4, then the number of uncovered cells *C*_*uc*_ are the positive prime numbers $P^{+}$ after each increment, e.g., if (*S*_*n*_) = 5, then the number of uncovered cells is 2. We acknowledge that the result is based on observation using the plan in Figure 3. Example and code of the visualized measurement process have been added to the supplemental documents. The uniqueness of the Delaunay triangulation makes different DIMASS plans have different outcomes. An interesting part of the result is how the number of uncovered cells became structured in line with the sensor range (i.e., keep increasing in a structured pattern, e.g., 0, 1, 2, 3, and 5). In conclusion, the coverage for the agents depends on the planned path, and the result shows a certain level of structure in the number of uncovered cells.

### 5.2 Algorithms Complexity Performance Comparison

Table 9 describes the algorithms’ complexities performance comparison, which could be used in grading the computational and implementational efforts of the algorithms. From Table 9, DIMASS shows good performance in terms of time complexities (being linear) and cyclomatic complexities. The cyclomatic complexity of the fixed-pattern methods follows the authors’ implementation as provided in the supplemental document (a different implementation may give a different result). The authors tried their best to reduce the cyclomatic number during implementation (by avoiding unnecessary loops, functions, etc.). As such, a lower number can be achieved with a different implementation because there are many ways of implementing a task in object-oriented programming languages, especially the applied Java programming language (codes can be found in the supplemental documents).

**TABLE 9:** Cyclomatic complexity and time complexity performance comparison.

Algorithm	Cyclomatic Complexity	Time Complexity
DIMASS	2	*O*(*n*)
Lévy flight (Chawla and Duhan, 2018)	3	*O*(*n*)
Parallel track (Bevacqua et al., 2015; Jensen-Nau et al., 2021)	7	*O* (*n* ^2^)
Creep lining (Bevacqua et al., 2015; Jensen-Nau et al., 2021)	9	*O* (*n* ^2^)
Sector search (Bevacqua et al., 2015; Cabreira et al., 2019)	11	*O* (*n* ^2^)
Expanding squares (Bevacqua et al., 2015; Cabreira et al., 2019)	7	*O* (*n* ^2^)
Zamboni search (João, 2012)	7	*O* (*n* ^2^)

### 5.3 Qualitative Metrics Performance

This subsection discusses the qualitative metrics performance comparison of the algorithms.

#### 5.3.1 Scalability

Definition 3.2, Definition 3.3, Proposition 1, and control rules for initial seeds waypoints demonstrate the possibility of applying Algorithm 1 (DIMASS) to multiple agents in a scalable manner. For instance, rules can be applied to control the initial waypoints and overall plan variation for multiple agents without agents’ interaction, e.g., using Definitions 3.2 and 3.3, as shown in Figure 4. As such, multiple agents’ solution requires a lower computational demand, number of agents’ interaction, energy (i.e., information exchange consumes power), mission time (due to absence of interaction), etc., based on the algorithm complexity of Table 9. The DIMASS has linear complexity (from Table 9) because a plan for *n* number of agents can be generated at once when distinct protocols (rules) are specified. That is, agents can generate their plans independently. Similarly, the Lévy flight approach (Chawla and Duhan, 2018; Sutantyo et al., 2011) can be considered as scalable because agents generate their plans independently (i.e., the plan efficiency is controlled by the Lévy distribution). However, the coordination of agents is challenging due to the pseudo-randomness of the waypoints’ generation process. On the other hand, structuring the fixed-pattern methods is quite challenging. This would require each agent’s plan consideration and revision, which increases the time complexity and makes scalability worse. For instance, assume the implementation of a parallel track (implementation picture is available in the supplemental documents folder) for four UAVs, track balancing is quite challenging due to inflexibility. The effective structuring of paths and tracks is challenging because it possesses a fixed structure, and the level of the challenge increases based on the number of agents. The same challenge happens with other methods such as parallel track, Zamboni search, and expanding squares (Cabreira et al., 2019; Bevacqua et al., 2015; João, 2012). For example, the challenge could be during section segmentation (i.e., vertically or horizontally, as described by the parallel track scalability picture of the supplemental document). Thus, the associated metrics for scalability are the cyclomatic and time complexities of Table 9 with respect to the resource utilization results of Tables 8 and 7. That is, the lower, more stable, and linear the time and cyclomatic complexities, the higher the scalability, and DIMASS shows a good performance.

#### 5.3.2 Adaptability

The ability to adjust the waypoints angles, edges, and projecting quadrants of DIMASS (Algorithm 1) supports its adaptability. For instance, it provides a unique way of monitoring the area coverage tasks, e.g., directing agents’ final waypoints close to the charging points when needed, searching for the best solution, controlling location’s number of visits using probabilities, detected fire (target) mapping, roads mapping, etc. For example, assume UAV4 (A4) from Figure 1 detects the fire O1 (based on the sensor state), the fire mapping task could be a set of short range waypoints in terms of angles (*θ*_*i*_), quadrants (*Q*_*i*_), and edge (*E*_*i*_) length to map the fire shape. That is, the transition of the agents’ belief and action configuration is modeled as *O* : *γ*_*i*_ × *S*_*condition*_ × *δ*_*i*_ → *w*_*i*_ (*θ*_*i*_ × *Q*_*i*_ × *E*_*i*_) (all symbols were defined in Section 3). In other words, the choice of angle, edges, and quadrant size is defined jointly by the agent’s situation (sensor state, i.e., belief and location) and environmental condition, e.g., the PC information on wind speed, wind direction, etc., which determine the fire (target) spread rate. For instance, the fire spread rate increases with an increase in wind speed. As such, the mapping task can be a zig-zag path of the locations with fire presence and absence continuously, i.e., a series of waypoints with a configuration of when a fire is detected until when it is absent. Similarly, considering the scene in Figure 1, when a road detecting agent finds its target, it would track that road by tilting its sensor within a certain radius, edge lengths, quadrants, and angles. Probability could be assigned to interesting k-previous waypoints (past waypoints), e.g., junctions, such that when the agent finishes its current task, it will return to the location and continue from there. The locations probabilities marks could be stored in the agent’s short term, medium- or long-term memories based on the saliency of the waypoint. For example, if the available detected roads could give a good evacuation plan, then another road tracking could be marked as non-important by lowering its junction’s probability value until the exit of the critical situation. This process resembles the operation of simulated annealing in terms of memory categorization and probability assignment; and smart Rapidly-exploring Random Tree (RRT) in terms of radius assignment (Nasir et al., 2013; Varty, 2017). That is, the projection angle, quadrant, and edge are based on the road detection radius and inclination towards the direction of the road. Lévy flight lacks adaptability due to the pseudorandom waypoint generation (Chawla and Duhan, 2018; Nurzaman et al., 2009). The structure of the fixed-pattern methods (square, rectangular and triangular shapes) affects their adaptability, e.g., during narrow space exploration. Thus, Algorithm 1 (DIMASS) can be extended to handle different forms of tasks, e.g., mapping using different waypoints’ edges, angles, and quadrant configurations. For the Lévy flight method, waypoints controlled from pseudorandom number generation is difficult or even impossible due to the standard deviation values of Tables 7 and 8. The fixed-pattern methods geometric shapes affects their adaptability (e.g., assume applying any of the fixed-pattern, e.g., parallel track, sector search, etc., search for road tracking). This will be difficult unless full search space coverage is needed. Thus, we propose the number of path control elements (quadrant, edges, and angles) as the measure for comparing the adaptability of the algorithms as described in Table 10.

**TABLE 10:** Algorithms adaptability comparison.

Algorithm	Controllable path elements	Controllable elements	Comments
DIMASS	3	\{quadrants, angles, edges\}	All variables can be controlled
Lévy flight (Chawla and Duhan, 2018; Sutantyo et al., 2011)	0	*¬*\{quadrants, angles, edges\}	None of the variables can be controlled
Parallel track (Bevacqua et al., 2015; Jensen-Nau et al., 2021)	1	\{*¬*quadrants, *¬* angles, edges\}	Edges can be controlled, whereas angles and quadrants are fixed because angle has to be either 90° or 180°
Creep lining (Bevacqua et al., 2015; Jensen-Nau et al., 2021)	1	\{*¬*quadrants, *¬* angles, edges\}	Edges can be controlled, whereas angles and quadrants are fixed because angle has to be either 90° or 180°
Sector search (Bevacqua et al., 2015; Cabreira et al., 2019)	2	\{*¬*quadrants, angles, edges\}	Changing quadrants configuration of sector search will make it not to be in sector form anymore
Expanding squares (Bevacqua et al., 2015; Cabreira et al., 2019)	1	\{*¬*quadrants, *¬* angles, edges\}	Edges can be controlled, whereas angles and quadrants are fixed because angle has to be either 90° or 180°
Zamboni search (João, 2012)	1	\{*¬*quadrants, *¬* angles, edges\}	Edges can be controlled, whereas angles and quadrants are fixed because angle has to be either 90° or 180°

Table 10 describes the algorithms’ adaptability measures using angle, quadrants, and edges customization as the measuring metrics. Therefore, based on the result in Table 10, DIMASS has the highest number of controllable path elements (angles, quadrants, and edges length).

#### 5.3.3 Predictability

From the fixed-pattern methods of the supplemental document and the DIMASS (Algorithm 1), agents’ locations can be estimated based on their speed and the plan generation rules. For example, from Figure 3, if a UAV is starting from waypoint W1 with a speed of 30 m/s, assume the length between waypoints W1 and W2 is 5.6 km; then, at the end of its second minute, it is expected to be 3.6 km away from the initial point (i.e., 2 × 60 × 30/1,000). Note that other reports could be incorporated, e.g., upwind and downwind acceleration and retardation. Thus, the predictability feature would help data collection (by arranging rendezvous among agents) and failure recovery (by estimating agents’ location). Additionally, the predictability feature could help in structuring the agents’ sensing range either periodically (after every time threshold) or waypoint-based. For the Lévy flight approach, predictability is difficult based on the standard deviation values of Tables 7 and 8, while fixed-pattern methods and DIMASS are predictable. One could say the possible solution to improve the predictability of the Lévy flight is to limit the seed of the random numbers. Interestingly, limiting the range of the random number seeds for Lévy flight has no effect on its predictability, as described by the results in Table 11. The values were taken from the average of 10 different AMASE experiments.

**TABLE 11:** Effect of changing the Lévy flight random number seeds on predictability.

#	Random number seed range	Avg. Path divergence *km* ^2^ *μ* (*σ*)	Avg. Number of waypoints (*σ*)
1	1–5	273.58 (73.34)	7.6 (1.16)
2	1–10	288.39 (61.75)	10.8 (1.90)
3	1–15	260.34 (94.42)	6.2 (1.46)
4	1–20	275.86 (119.35)	7.4 (1.58)
5	1–25	193.87 (43.64)	5.1 (0.64)
6	1–30	293.29 (83.3)	7.8 (1.64)

Table 11 shows that limiting the Lévy flight random number seeds range has no effect on its predictability even with the least range of 1–5 numbers. For example, from Table 11 #1, the range 1–5 has higher path divergence than #3 with range 1–15 and #4 with range 1–20. Thus, the number of waypoints and path divergence demonstrates no relation with the random number seeds ranges based on the mean and standard deviation values in Table 11. This means predictability of Lévy flight is difficult to achieve through random number seeds control.

## 6 Discussion

The experiment compared DIMASS with examples of existing methods across qualitative and quantitative metrics. The results in Tables 8–9 show that the DIMASS delivers a solution with good coverage, path divergence, time complexity, cyclomatic complexity, redundant search, adaptability, predictability, and scalability, despite being the non-best solution, i.e., the evaluation used fixed protocols in which shorter edges are half of the longer ones from Figure 3. The DIMASS best solution can be obtained by stretching the edges, angles, and quadrants through searching and information exchange until the best solution is found (i.e., a solution that best utilizes the evaluation metrics). For example, the number of visits to a cell can be managed using probabilities, i.e., an increase in the probability of the cell after each visit. This will allow waypoints adjustment (i.e., edges, angles, and quadrants adjustment) based on cells’ number of visits probabilities. The redundant search comparison considers two UAVs because the higher the number of UAVs, the higher the redundant search (Koenig and Liu, 2001; Li et al., 2011). The predictability supports the estimation of agents’ current and future locations, which in return supports data collection rendezvous between PCs and micro UAVs; and promotes failure recovery (i.e., by estimating the location of the UAV based on speed and environment exogenous variables, e.g., wind speed, wind direction, etc.). In terms of scalability, DIMASS seed waypoints generation and the uniqueness of the waypoint generation rules [based on the Delaunay triangulation uniqueness (Cignoni et al., 1998)] ensure a scalable solution (i.e., there is no need for revising all the agents’ plans when seeds waypoints and rules are different). Thus, this supports independent agents’ planning. As such, the uniqueness of the plan and policy variation ensures stable resource demand (measured based on the running time and cyclomatic complexity) for a larger number of UAVs, as described in Figure 4.

**FIGURE 8:**
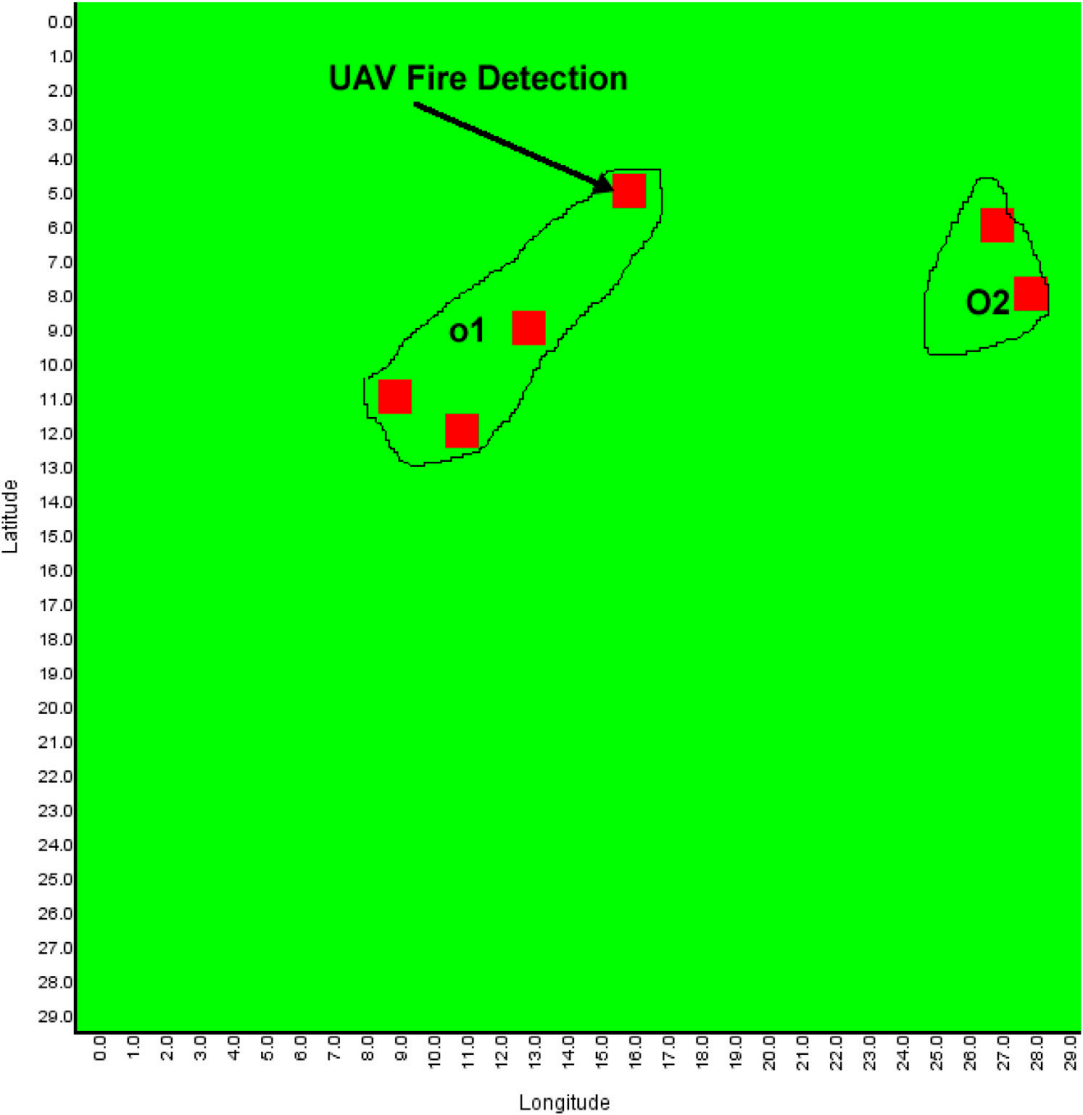
Example of one of the UAVs fire detection presentation using a heat map.

Adaptability will be maintained by changing the projection angles, quadrants, and edges of a plan. For example, target (fires) tracking would be done by changing the angles, edges, and quadrants based on the known dynamic variables (e.g., tilting sensors toward the expected fire direction based on wind speed). Based on observation and analysis of the outlined methods, most of the fixed-pattern methods are highly structured and have poor adaptability. For example, parallel track, creeping line, expanding squares, Zamboni, and sector methods (Bevacqua et al., 2015; Cabreira et al., 2019; João, 2012) as described in the supplemental documents folder, all follow a fixed structured geometric pattern and explore the environment sequentially. However, sector search is similar to DIMASS in terms of adaptability. For example, from the sector search figure of the Supplemental document, the angle and projection edges can be changed to incorporate multiple agents. Despite the potential for adaptability, sector search lacks the following features in comparison with the DIMASS. Sector search follows geometric shapes, which make them inappropriate for narrow space exploration, e.g., road mapping, whereas, in DIMASS, policies are used to control the path projections. Thus, adaptability is limited in sector search, as described in Table 10. Similarly, in Voronoi tessellation, agents visit the centres of the circumcircles of the Delaunay Triangulation (Hasegawa et al., 2012; McLain et al., 2001; So and Ye, 2005). This approach resembles the initial version of our algorithm in Appendix 1, with the absence of layering. In conclusion, DIMASS has some similarities with sector search and Voronoi tessellation but provides superior performance across the outlined metrics. In terms of sensor information presentation, say to the Subject Matter Experts (SMEs), the search space can be segmented into equal cells with target detection highlighted by a different colour, i.e., red for fire, green for covered cells (Figure 8). Figure 8 describes a heat map presentation for UAV 4 of Figure 4 using 900 (30 by 30) equals cells, i.e., *S* = {*s*_1_, *s*_2_, *s*_3_, ..., *s*_*900*_}. Each fire detection at a particular location (cell *S*_*i*_) is marked with a different colour. Therefore, the heat map will produce a picture of the situation of the environment at the PC or host levels.

### 6.1 Moving From Simulation to Reality

The implementation of DIMASS on real UAVs is simple. The process starts by selecting the seeds waypoints (e.g., the longest non-cross waypoints in Figure 3), and then a function can be developed to generate the remaining waypoints by taking some parameters, e.g., using the function *generateWaypoint* (*L*_*x*_, *L*_*y*_, *e*, *q*, *θ*, *h*, *n*), where *L*_*x*_ and *L*_*y*_ are the longitude and latitudes of the current waypoint, *e* is the edge length, *q* is the projecting quadrant (first to fourth), *θ* is the projecting angle, *h* is the height of the waypoint (e.g., to avoid collision), and *n* is the number of waypoints in a layer of the MAP/DULAR solution (based on the Delaunay triangulation theorems discussed). In other words, the function *generateWaypoint* (*L*_*x*_, *L*_*y*_, *e*, *q*, *θ*, *h*, *n*) produce a waypoint based on the current waypoint, opposing edge, angle (*θ*), number of waypoints in a layer, and the implemented rules as described in Section 3. Waypoints latitudes and longitudes distance differences (i.e., for *e*) can be computed using the Haversine formula or Euclidean distance can be used for planar coordinates. This can be implemented in any programming language (e.g., Java, as described in the supplemental documents).

**FIGURE 9:**
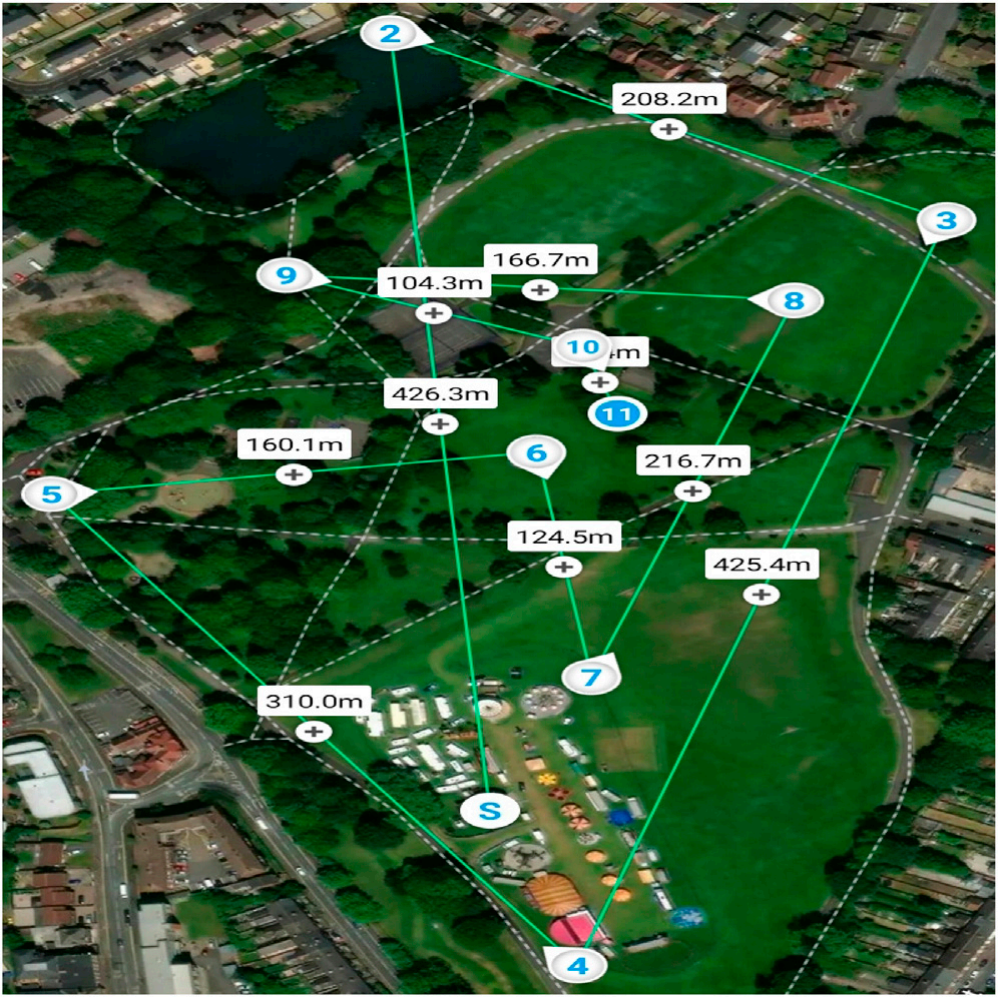
Implementation of algorithm DIMASS on real drone android application (DJI Pilot app).

The generated plan can be transferred easily to the UAVs using the respective UAVs’ (drone’s) mobile application (downloaded from either Google play store or Appstore), e.g., DJI GO, DJI Pilot, FreeFlight 6, FreeFlight Pro, etc., for DJI and Parrots drones. Before testing the proposed algorithm on real UAVs, the authors acquired the operator and flyer identification numbers (drone flying licenses) from the Civil Aviation Authority (CAA) of the United Kingdom. The flying took place in one of the areas of Birmingham in the United Kingdom, as shown in Figure 9, i.e., the real UAV flying experiment took place in the United Kingdom, whereas the fire spread experiment was conducted in Nigeria. Figure 9 shows the implementation of DIMASS on the DJI Pilot mobile app to explore a search space (forest) starting from waypoint S to 11 (just like Figure 3). The plan creation and modification (i.e., quadrants, edges, and angles configurations) occurs simply by clicking and dragging waypoints. The displayed distance between waypoints helps in defining edge length (as described in Figure 9). In addition, the mobile apps allow plans storage and deletion. Thus, the plan storage will allow routine lookout planning for the team of UAVs (i.e., waypoint plans can be saved and utilized for routine area searching, e.g., routine forest fire searching). Alternatively, waypoints can be sent to the UAVs via python code for the programmable drones, e.g., DJI Tello Edu python application programming interface (API)^4^. For larger UAVs, e.g., DJI matrices 100, onboard computers can be mounted to perform information analysis and other complex tasks (Alexis, 2019).

The authors flew three UAVs, Parrot Bebob 1 and 2 and DJI Phantom 3. Each UAV has a controlling app running on a tablet (one tablet per UAV). The outcome is similar to the simulation results as described in Figure 4. Thus, the proposed DIMASS is easy to implement on real devices.

### 6.2 Limitations

While we have demonstrated good performance of DIMASS, both in simulation and real UAVs, there are a number of limitations to explore in further work:

• Seed waypoints (first layer waypoints) need to be defined systematically. In the case of a large number of UAVs, the seed waypoints definition function must be systematic. For example, using four UAVs (Figure 3) is less challenging than 50 UAVs unless rules grouping is applied (i.e., a group of UAVs will be using different control protocols with others). Thus, in a large team of UAVs, seed waypoints control will be challenging. Therefore, layers configuration and control policies will be difficult. Although this scalability issue can be solved by using waypoints altitudes variations, Definition 3.2, Definition 3.3, Proposition 1, and seed waypoints variation, any non-organized set of plans could cause team disorganization (poor coordination). Thus, we agree that the type of search task used for our use case would be best performed by a team of around four to seven UAVs which are controllable by a single expert (Baber et al., 2011).
• For a large number of layers and agents (e.g., UAVs), searching for the best solution could demand large computational resources, which must be bounded, e.g., by setting a number of iterations to avoid plan delay. For instance, configuring the best edges for 10 UAVs given 50 seed waypoints could require a lot of information exchange among UAVs. Therefore, large consumption of memory, communication bandwidth, and processing power will likely occur at the mission preparation stage.
• Policies for controlling plan updates need to be defined. As such, in the case of a large number of UAVs, this will be quite challenging to control.
• Collision avoidance requires Detect and Avoid (DAA) techniques using sensors or some form of organization, e.g., stop and pass rules (i.e., stop and wait for rules to avoid collision), waypoints altitudes variation, etc. Again, implementing effective collision avoidance for a large number of UAVs and waypoints will be difficult.

## 7 Conclusion

We introduce a novel hybrid multiagent search algorithm for a team of UAVs under destination uncertainty and limited agents’ resource constraints. The algorithm used mathematically derived rules from the Delaunay triangulation process to control agent waypoints’ efficient generation. The algorithm promotes scalability, adaptability, predictability, and resource utilization of the team of UAVs tasked to conduct the search mission. The performance of the proposed algorithm was evaluated on a multi-UAV mission for forest fire searching. Results proved an efficient solution as described in Section 5. We believe that the proposed algorithm is an advancement of the fixed-pattern and pseudorandom methods. We suggest that instead of tasking the agents with complex operations through communication and messages processing during a search mission, we can make their tasks easier by applying simple control rules. In addition, DIMASS demonstrates easy implementation on real UAVs. Future work will look at how the proposed algorithm will support a large number of UAVs, agents decision-making, effective sensor information collating process for a bigger team, agents control through learning, and more path control theorems.

## Data Availability

The original contributions presented in the study are included in the article/Supplementary Material; further inquiries can be directed to the corresponding authors.

## Footnotes

1
https://github.com/afrl-rq/OpenAMASE

2
https://www.gov.uk/government/publications/unmanned-aircraft-systems-jdp-0-302

3
https://github.com/code4funSydney/Tello

4
https://github.com/code4funSydney/Tello

## Appendix 1: Fixed-pattern (geometric-patterns) approaches

This is the result of the initial version of DIMASS.

Algorithm A1 The Delaunay triangulation–based MAP/DULAR algorithm.

**TABLE A1:** Algorithm A1 performance.

Metric	Value
Coverage	1
Path divergence	834.83 km^2^
Cyclomatic complexity	19
Running time	*O* (*n* ^2^ *logn*)
Redundant search: sensor range:*r*_*v*_	Number of waypoints A1(r), A1(R)
*r*_*v*_ = 5%	0,0
*r*_*v*_ = 10%	1,4
*r*_*v*_ = 15%	5,2
*r*_*v*_ = 20%	5,3
*r*_*v*_ = 25%	5,3
*r*_*v*_ = 30%	6,3
*r*_*v*_ = 35%	6,4
*r*_*v*_ = 40%	6,5
*r*_*v*_ = 45%	6,5
*r*_*v*_ = 50%	6,6
