# Supplementary material for: DIMASS: A Delaunay-Inspired, Hybrid Approach to a Team of Agents Search Strategy
Source: Front Robot AI. 2022 Jun 29;9:851846. doi: 10.3389/frobt.2022.851846 (PMC9277356; doi:10.3389/frobt.2022.851846)
Supplement: Supplementary file 2 [file DataSheet1.PDF]

Algorithms Application Procedure

This is a short description on how the proposed algorithms can be applied for area coverage operation, e.g., during search and rescue, disaster management, surveillance mission, etc. The following steps describe the application procedure.

1. Define the seed waypoints generation strategy, for example, the longest non-crossed jumps as in the paper, waypoints reflection, refraction, or any suitable strategy.
2. Define the waypoints projection angles, edges and quadrants rules for algorithm 2 or the Delaunay triangulation process for algorithm 1. The authors applied the Delaunay triangulation plugins for Matlab (https://www.mathworks.com/help/matlab/ref/delaunay.html), Python (https://towardsdatascience.com/delaunay-triangulation-228a86d1ddad), and Java (https://github.com/themadcreator/delaunay/blob/master/src/org/delaunay/algorithm/Triangulation.java). For other programming languages, the applicant can import it from their respective platforms or simply uses the attached Matlab script and then import the output. For real-UAVs, the path can be drawn as waypoints or specified the coordinates of the waypoints (as done by the authors on Ryze Tello Edu).
3. Define the angle, edge, and quadrant generation rule according layer.

Additional Propositions and Theorems

Due to the brevity of the submission, propositions and theorems on the angle choice, shortest paths, and swarm control were omitted. This will be available in the future journal.

Demonstration Videos and Codes

Attached with this supplemental report are the videos, AMASE simulation XML code, and codes for the proposed solutions.
